# Gold nanolayer and nanocluster coatings induced by heat treatment and evaporation technique

**DOI:** 10.1186/1556-276X-8-249

**Published:** 2013-05-22

**Authors:** Anna Schaub, Petr Slepička, Irena Kašpárková, Petr Malinský, Anna Macková, Václav Švorčík

**Affiliations:** 1 Department of Solid State Engineering, Institute of Chemical Technology, Prague 166 28, Czech Republic; 2 Nuclear Physics Institute, Academy of Sciences of the Czech Republic, Rez, Czech Republic; 3 Faculty of Science, J.E. Purkyně University, Ústí nad Labem, Czech Republic

**Keywords:** Glass substrate, Gold coating, Nanostructure, Surface properties, Thermal annealing

## Abstract

The paper is focused on the preparation and surface characterization of gold coatings and nanostructures deposited on glass substrate. Different approaches for the layer preparation were applied. The gold was deposited on the glass with (i) room temperature, (ii) glass heated to 300°C, and (iii) the room temperature-deposited glass which was consequently annealed to 300°C. The sheet resistance and concentration of free carriers were determined by the van der Pauw method. Surface morphology was characterized using an atomic force microscopy. The optical properties of gold nanostructures were measured by UV–vis spectroscopy. The evaporation technique combined with simultaneous heating of the glass leads to change of the sheet resistance, surface roughness, and optical properties of gold nanostructures. The electrically continuous layers are formed for significantly higher thickness (18 nm), if the substrate is heated during evaporation process. The annealing process influences both the structure and optical properties of gold nanostructures. The elevated temperature of glass during evaporation amplifies the peak of plasmon resonance in the structures, the surface morphology being significantly altered.

## Background

Nanostructured thin films play nowadays a quite significant role in various material science and technology applications. In particular, a considerable attention has been drawn to the structure and properties of thin metal films deposited on non-metal surfaces due to their attractive applications in electronic, magnetic, and optical devices [1]. Gold nanolayers are perspective structures for certain applications due to their unique electrical and optical properties. Gold in the form of thin films is nowadays used in a vast range of applications such as microelectromechanical systems and nanoelectromechanical systems, sensors and electronic textiles, bioengineering, as a generator of nonlinear optical properties, or in devices for surface-enhanced Raman scattering [2-4].

Layers consisting of gold nanoparticles (AuNP) are usually prepared by precipitation from aqueous solutions on various materials, e.g., on etched glass surfaces. The thermal annealing of thin gold films produced by thermal evaporation or sputtering can also lead to a disaggregation into particles [1,5,6]. The formation of AuNP from continuous gold layers is driven by the minimization of surface energy and is denoted as solid-state dewetting. All the described methods suffer from the poor adhesion of AuNP to the substrate surface [7]. The electrical resistance measurement shows that the nanoparticles are conductive even at a small metal volume fraction. Due to the aggregation effect, the optical transmission spectra exhibited an enhanced transmission band around 500 nm arising from the surface plasmon resonance. Many authors have developed theories of distortion of crystalline lattice in nanostructures, some of them being applicable on nanoparticles [8-11]. Spherical nanoparticles surrounded ‘by air’ have different behaviors as nanostructures deposited on solid surface [12,13].

This work is focused on glass substrate and subsequent deposition of Au layer by evaporation. The gold deposition was carried out at room temperature (RT) and at 300°C. Then the samples prepared on the substrate at room temperature in this way were annealed at 300°C. The effects of annealing or deposition on glass substrate with elevated temperature were studied using atomic force microscopy (AFM, for surface morphology and roughness), UV–vis spectroscopy and electrical measurements (for sheet resistance and volume-free charge carrier concentration). The novelty of this research lies in the precise simultaneous study of nanostructures induced by evaporation on heated and non-heated glass substrate and its comparison to subsequently annealed structures. The optical and electrical characterizations connected with the changes in surface morphology induced by the particle surface diffusion bring important new information to this field of research.

## Methods

Glass substrate (Menzel-Glaser, Braunschweig, Germany) with the dimension 20 × 20 mm^2^ was used for the present experiments. Vacuum evaporation was performed on Leybold-Heraeus, Univex 450 device (Oerlikon Leybold Vacuum GmbH, Cologne, Germany) with typical parameters: room deposition temperature, total pressure of about 2.10^−5^ Pa, molybdenum container with source current >5 A. The gold deposition was accomplished at room temperature (25°C) and at 300°C (pressure of 2 × 10^−5^ Pa) using gold target (purity 99.99%, supplied by Goodfellow Ltd., Huntingdon, Cambridgeshire, UK). The thicknesses of the deposited Au were determined from AFM analysis and were in intervals of 2 to 40 nm. The post-deposition annealing of the gold/glass samples was carried out in air at 300°C (±3°C) for 1 h using a thermostat Binder oven (Binder GmbH, Tuttlingen, Germany). The annealed samples were left to cool in air to room temperature.

For the sheet resistance and concentration of free charge carrier determination of Au layer evaporated onto glass, the van der Pauw method was used. The measurement was accomplished with direct current (dc) and a homogeneous dc magnetic field, with a polarity commutation of both quantities. Keithley 2400 (Keithley Instruments Inc., Cleveland, OH, USA) served as a source of constant current. The voltage response was measured with Keithley 2010 multimeter. The magnetic field (*B* = 0.4 T) was generated by an electromagnet fed from the Keithley 2440 source. The computer code, working under the LabView 8.5 system (National Instruments, Austin, TX, USA), was used for the experiment control and data evaluation [14]. The values have been obtained from the six independent measurements under previous set-up, and the average values have been introduced in the graph of sheet resistance and the free carrier concentration with error of measurement not exceeding 5%. The values of sheet resistance have been also confirmed by the modified two-point technique [14] as an alternative method for sheet resistance determination.

The surface morphology of glass and Au-metalized glass was examined using AFM in tapping mode under ambient conditions with a CP II Veeco microscope (Bruker Corp., Santa Barbara, CA, USA). An etched Si probe (doped with P), RTESPA-CP, with spring constant of 20 to 80 N m^−1^ was used. The average mean roughness (*R*_a_) represents the arithmetic average of the deviations from the center plane of the samples. All samples have been measured repeatedly at three different areas on two samples; the error in the surface roughness measurement did not exceeded 7%.

The UV–vis spectra were measured using a PerkinElmer Lambda 25 spectrometer (PerkinElmer Inc., Waltham, MA, USA) in the spectral range from 330 to 1100 nm. Rutherford backscattering (RBS) analyses were performed on Tandetron 4130MC accelerator (Center of Accelerators and Nuclear Analytical Methods, Nuclear Physics Institute of the ASCR, Řež, Czech Republic) using 1.7 MeV ^4^He ions. The RBS measurement was realized at the CANAM infrastructure. The measurements were performed in IBM geometry with incident angle 0°, and laboratory scattering angle of 170°. The typical energy resolution of the spectrometer was FWHM = 15 keV. The RBS spectra were evaluated using SIMNRA and GISA softwares.

## Results and discussion

### Electrical properties of Au structures

**Figure 1:**
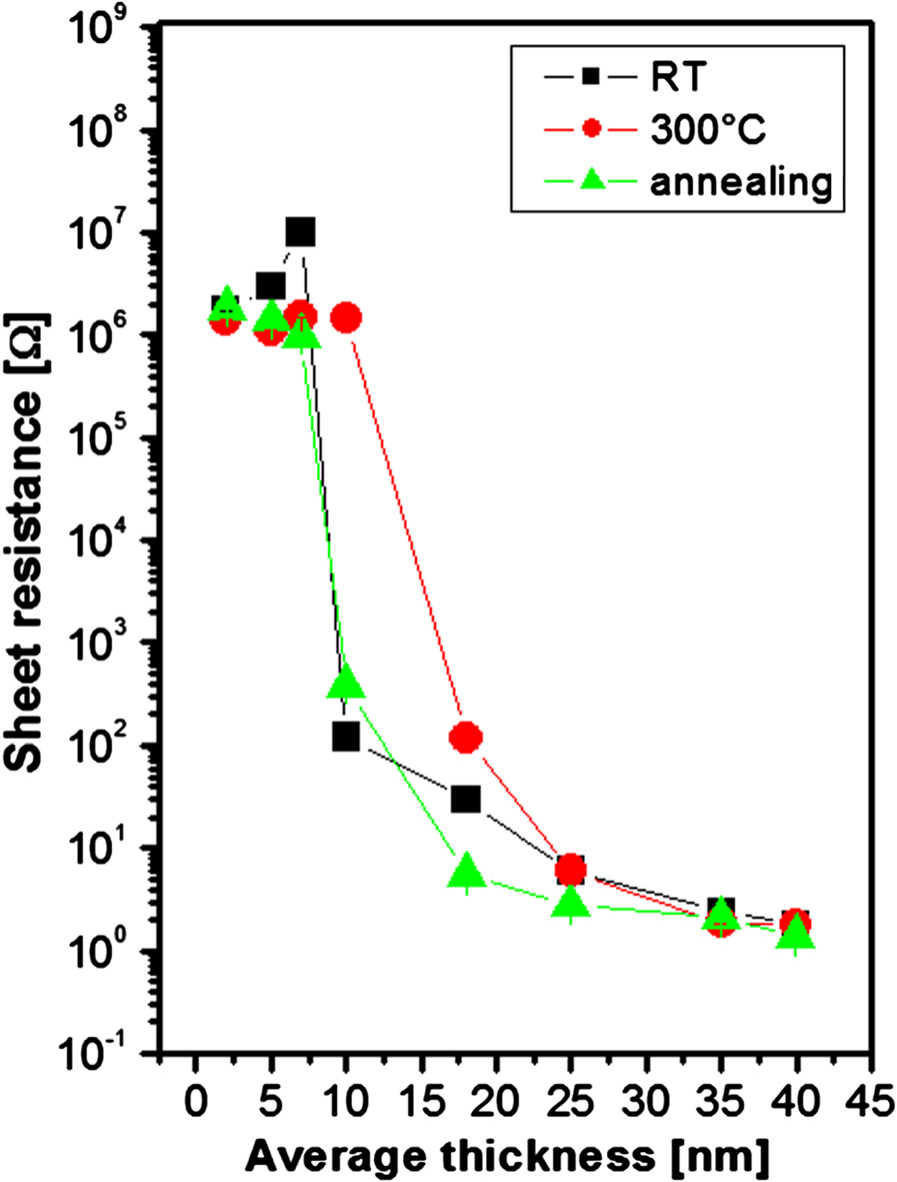
**Dependence of Au layer sheet resistance of evaporated samples deposited on glass at different temperatures.** The dependence of Au layer sheet resistance on the layer thickness measured for evaporated samples deposited on glass at room temperature (RT), deposited on substrate heated to 300°C (300°C) and deposited on glass with room temperature and consequently annealed at 300°C (annealing).

The dependence of the sheet resistance (*R*_s_) on the Au layer thickness is introduced in Figure 1. With increasing layer thickness, the *R*_s_ of the gold layer decreases as expected. The difference was found when the compared gold nanolayers evaporated on glass at room temperature and 300°C. The sharp decrease of the sheet resistance was observed (RT and annealing) for the thicknesses above 10 nm when an electrically continuous layer is formed. This is a rather different behavior from the sputtered Au nanolayers, when the formation of electrically continuous layer was shifted to higher thicknesses due to thermal annealing [15]. This is in contrast with the results obtained in this work for gold nanolayers deposited by evaporation. The threshold for the formation of electrically continuous layers is both for non-annealed and annealed nanolayers *ca.* 10 nm. This finding may be caused due to different adhesive force of gold prepared by evaporation in comparison to sputtering technique. Due to that fact the surface diffusion is suppressed, the local melting and mass redistribution are being probably preferred. A rather different situation was found for the layers evaporated on the glass, which is already heated to 300°C. Due to higher temperature of the glass during the deposition process, the surface diffusion takes place, which results in significant shift for the electrically continuous layer formation. The sharp decrease of sheet resistance was observed for the thickness *ca.* 20 nm (two times higher than for annealed one). The behavior of the layer deposited on heated glass (shift of the threshold for electrically continuous layer) is similar to those as-sputtered and then thermally annealed [15]. With further increase of the Au thickness, the pronounced decrease of *R*_s_ is observed, with the minimum being achieved for the thicknesses above 35 nm both for annealed Au and Au deposited on heated substrate (see Figure 1).

**Figure 2:**
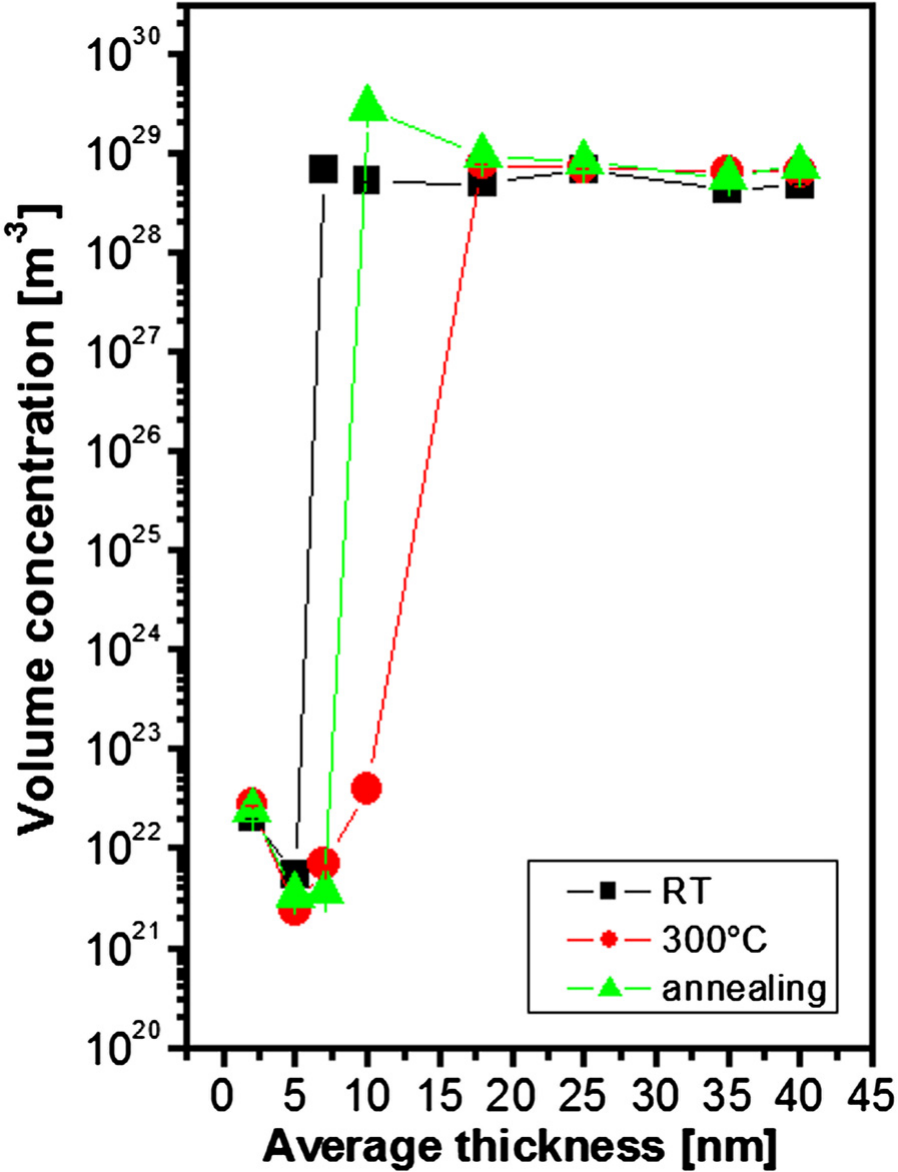
**Dependence of free carrier volume concentration in Au layer deposited on glass at different temperatures.** The dependence of free carrier volume concentration in Au layer on the layer thickness measured for evaporated samples deposited on glass at RT, deposited on substrate heated to 300°C (300°C) and deposited on glass with room temperature and consequently annealed at 300°C (annealing).

Free carrier volume concentration significantly affects the electrical conductance of materials. The dependence of the free carrier concentration on the Au layer thickness is shown in Figure 2. With the formation of an electrically continuous Au layer, the carrier concentration increases dramatically. The thickness for the transition to formation of electrically continuous layer is in a good correspondence with the measurement of *R*_s_ (see Figures 1 and 2). The sharp increase of free carrier concentration is shifted for the Au layers prepared by evaporation onto the heated substrate (300°C) to 20 nm which is in accordance with the results in Figure 1. The increase of free carrier concentration was observed in the layer thickness of 10 nm for the annealed Au layers and slightly lower thickness for the Au evaporated by room temperature. This minor difference can be caused by the different morphologies of Au nanostructures influencing the transport of free carriers in Au nanolayers after annealing, which will be discussed in the next chapter.

### Surface morphology

**Figure 3:**
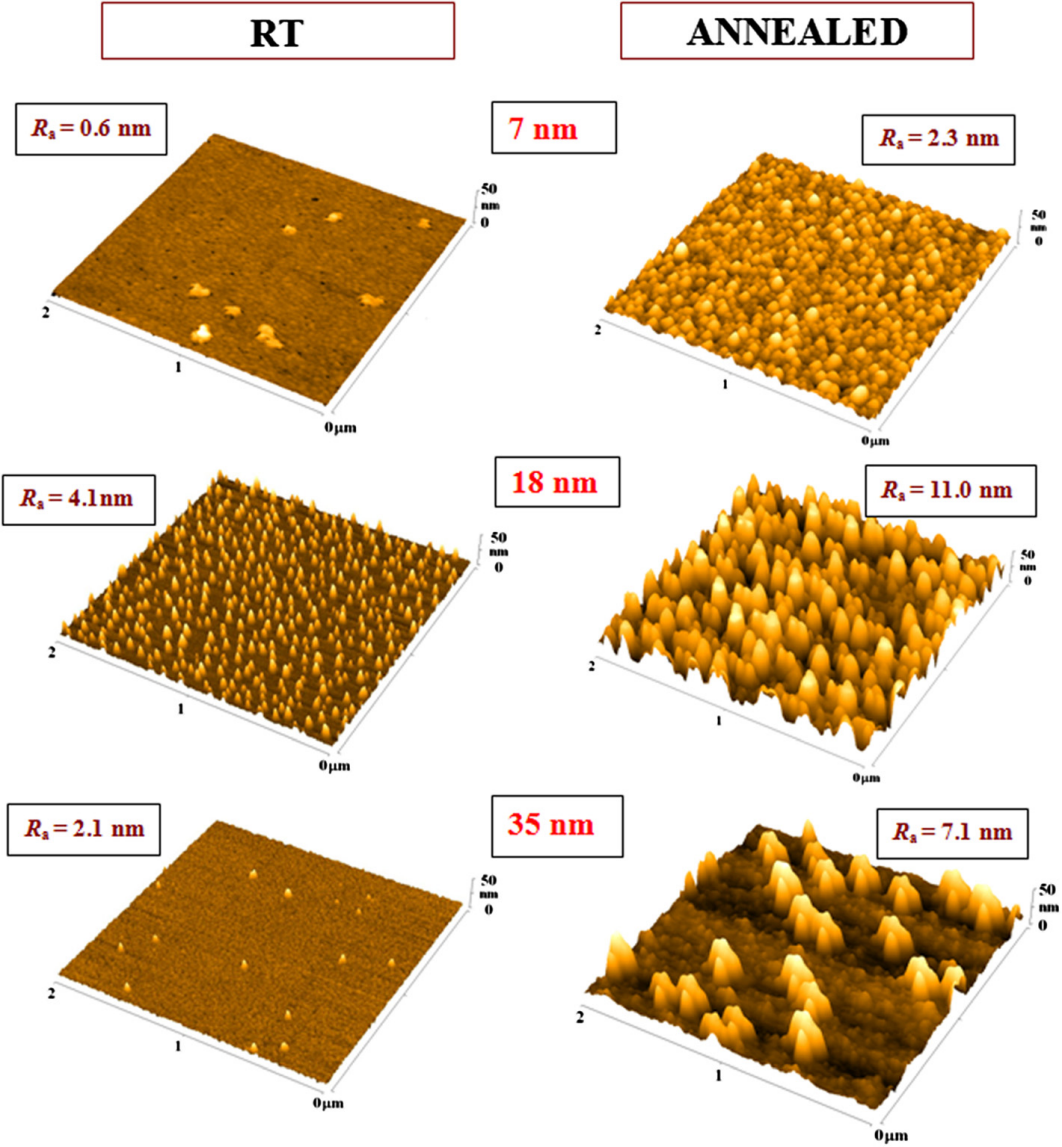
**AFM images of the evaporated Au layers at different temperatures.** AFM images of the evaporated Au layers on glass with room temperature (first column, RT) and the same samples consequently annealed at 300°C (second column, annealed). The thicknesses of evaporated Au were 7, 18, and 35 nm. *R*_a_ is the arithmetic mean surface roughness in nanometers.

The morphology of evaporated Au nanolayers of different thicknesses and their structures consequently annealed to 300°C is introduced in Figure 3. The surface morphology of electrically discontinuous (7 nm), electrically continuous (18 nm), and electrically continuous layer with minimum sheet resistance (35 nm) was chosen for the analysis. As it is obvious from Figure 3, the consequent thermal annealing leads to the significant increase of the surface roughness both for electrically continuous and discontinuous evaporated nanolayers. With increasing thickness of evaporated Au, the increase of surface roughness was observed first (first column, from 0.6 to 4.1 nm), and the globular structure appears on the glass. Further increase of Au thickness leads to the increase of layer’s homogeneity and the globular structure being less pronounced as well as the surface roughness. The thermal annealing leads to a significant increase of surface roughness (Figure 3, second column). The globular structure is strongly amplified probably due to the local surface melting of gold nanoparticles during the thermal annealing process [16]. The dimensions of globular structures are significantly higher in comparison to non-annealed ones. The surface morphology of the annealed Au with thickness of 35 nm is similar to those observed on glass substrate deposited by sputtering [15]. Similar changes in the morphology of the thin gold annealed structures and a sharp increase in surface roughness were observed on the samples annealed at 200°C for 20 h [17] and at 450°C for 2 h [18].

**Figure 4:**
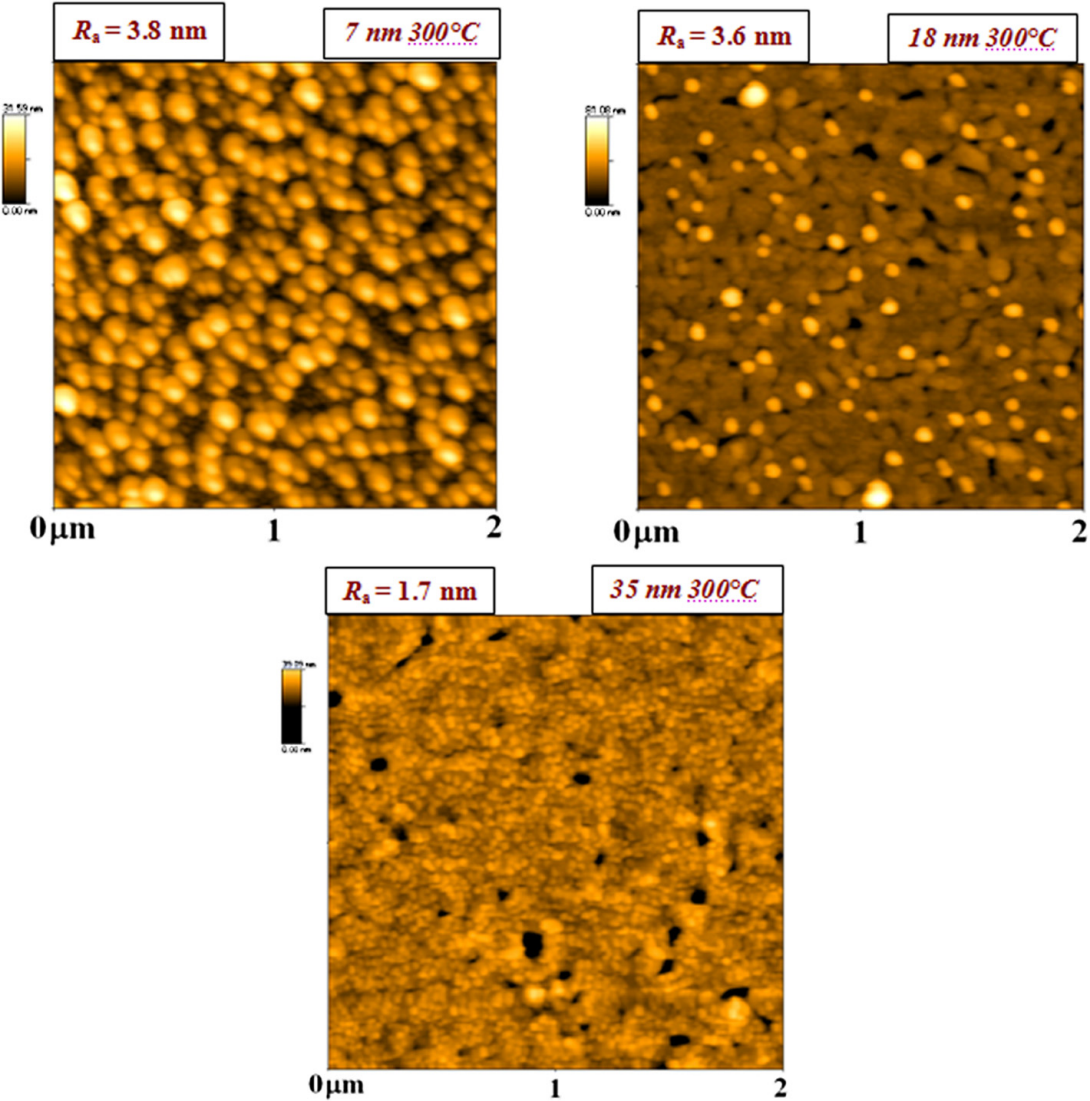
**AFM images of the evaporated Au layers on glass heated to 300°C.** The thicknesses of evaporated Au were 7, 18, and 35 nm. *R*_a_ is the arithmetic mean surface roughness in nanometers.

The rather different appearance of surface morphology was determined for evaporated Au deposited on glass already heated to 300°C (Figure 4). The gold layer of 7-nm thickness exhibited globular nanostructure with roughness of 3.8 nm. With increasing Au layer thickness, the globular nanostructure has a tendency to disappear. The electrically continuous nanolayer (35 nm) exhibits the lowest values of surface roughness (1.7 nm), the surface pattern being similar to those obtained for sputtered Au [19]. The reason for such appearance should be within the formation of nanolayer and its nucleation. The electrical measurement revealed that the difference in thickness when the electrically continuous layer (Figure 1) is formed for as-evaporated and consequently annealed layer and is minor in comparison to previously studied annealing of sputtered Au [5]. Therefore, we can suppose that the surface diffusion of gold nanoparticles is suppressed when the layer is heated, which is connected with the different surface wettability when the substrate is heated. The influence of surface diffusion may take place also in the case of evaporation in the already heated glass (Figure 4). The appearance of globular structures caused by the evaporation of 7-nm Au is probably caused by the surface melting of evaporated Au nanoparticles during the deposition process. Even when the melting process takes place, the surface diffusion is suppressed and the structure has regular and homogeneous character. With increasing thickness of gold nanolayer, the suppression of globular nanostructure is probably more pronounced by the decrease of temperature on the substrate due to ‘isolation’ caused by the increasing thickness of Au nanolayer. Therefore, the effect of surface melting is smaller and the structures are similar to those obtained for the samples evaporated on glass substrate under RT (Figure 3), with the roughness also being only mildly changed.

The influence of gold nanocluster formation has been also extensively studied [20] on mica. A phenomenological study was carried out to find a reliable way for the gold thin film preparation. The following parameters have been focused on: annealing time of the substrate before deposition of the gold film, deposition rate of the gold film, substrate temperature before and during evaporation and annealing time after the deposition [20]. Deposition of Au films on mica with the deposition temperature 500°C led to the similar structures that we achieved on glass heated to 300°C, where pores and whiskers have been observed [20].

The gold nanocluster formation on glass substrate is strongly influenced by the physical processes of vapor-deposited thin gold films on glass substrate [21]. The processes which can alter the layer’s growth may be, e.g., chemical or plasma modification of the substrate [21] or gold and glass wettability [21]. The bonds between the gold clusters and the glass substrates are usually weak, and their wettability is relatively bad. It was reported that the gold nuclei diffusion on the surface is increased, as well as their coalescence, when its wettability is poor [21]. On the contrary, if the wettability of gold for the substrate is improved (chemical modification of the surface), the interactions between the two materials are globally stronger, and both the diffusion and coalescence of the metal clusters are disfavored [21].

### Optical properties

**Figure 5:**
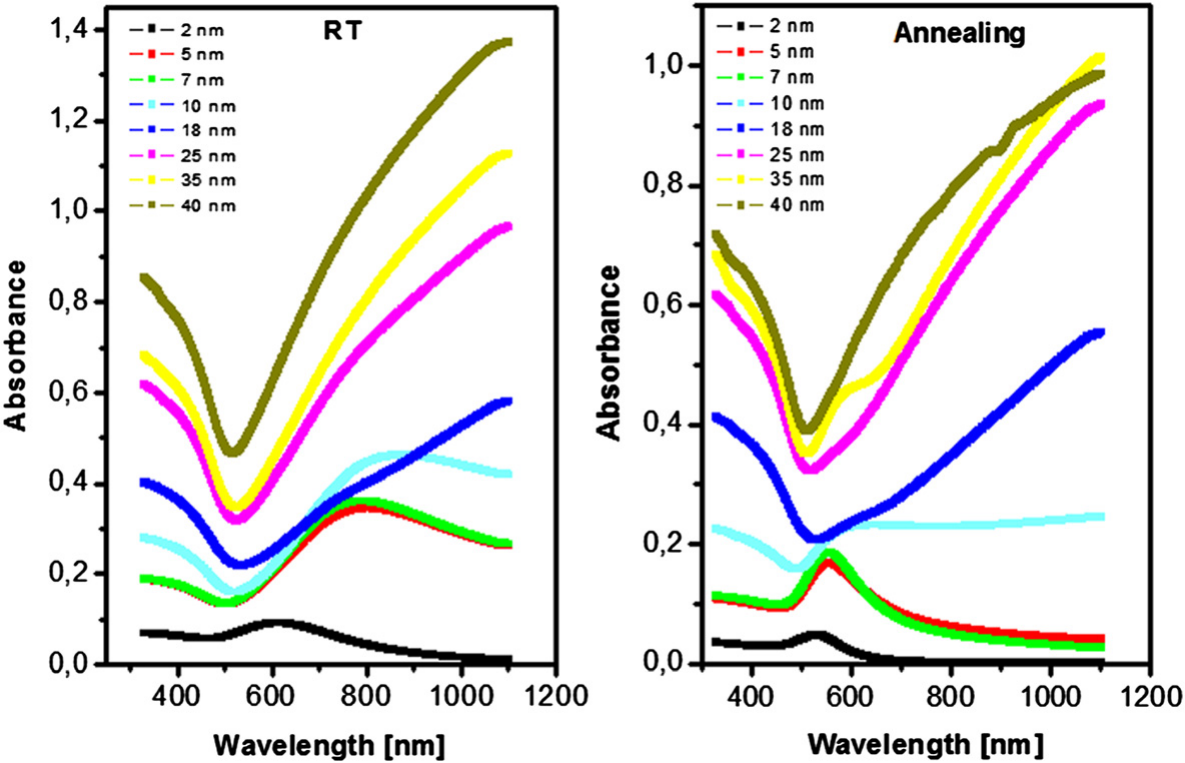
**UV–vis spectra of gold structures evaporated on glass - before (RT) and after annealing (annealing).** The numbers of the curves are Au thicknesses in nanometers.

The UV–vis extinction spectra of Au nanolayers deposited on substrate before and after annealing process are introduced in Figure 5. The absorbance of both annealed and non-annealed gold structures increases with increasing structure thickness as could be expected. From the comparison of the spectra of evaporated and annealed samples, it is seen that the annealed structures have qualitatively different shapes and lower absorbance. Both phenomena arise from structural changes due to annealing. From our previous experiments, which have been focused on the behavior of sputtered gold nanostructures on glass, it was determined that for the sputtered Au, a shift of 530-nm absorption peak was observed [5] which corresponds to surface plasmon resonance. This shift with increasing Au thickness towards longer wavelengths was probably related to the interconnection and mutual interaction of gold nanoparticles in the structure [5]. Similar shifts of the optical absorption peak were also observed during the reduction of gold sulfide particles (Au_2_S) to gold particles [22]. It was previously found that by controlling the initial size of the gold sulfide particles, the resonance shift can be correlated with a theoretical model that includes both quantum confinement and the resonance effects (the so-called surface plasmon resonance) [22]. Ultra-smooth surfaces from template-stripping procedures can be also used for periodic structures preparation [23], which can induce effects of surface plasmon resonance. The behavior of annealed gold nanolayers prepared by evaporation is rather different. The peak of plasmon resonance can be found for the annealed samples of thicknesses up to 7 nm (see Figure 5). In addition, the shift of the peak of plasmon resonance towards higher wavelengths as described earlier [5] was observed. The suppressed diffusion of the evaporated gold nanolayers during the annealing process may be the leading cause in the plasmon peak appearance.

**Figure 6:**
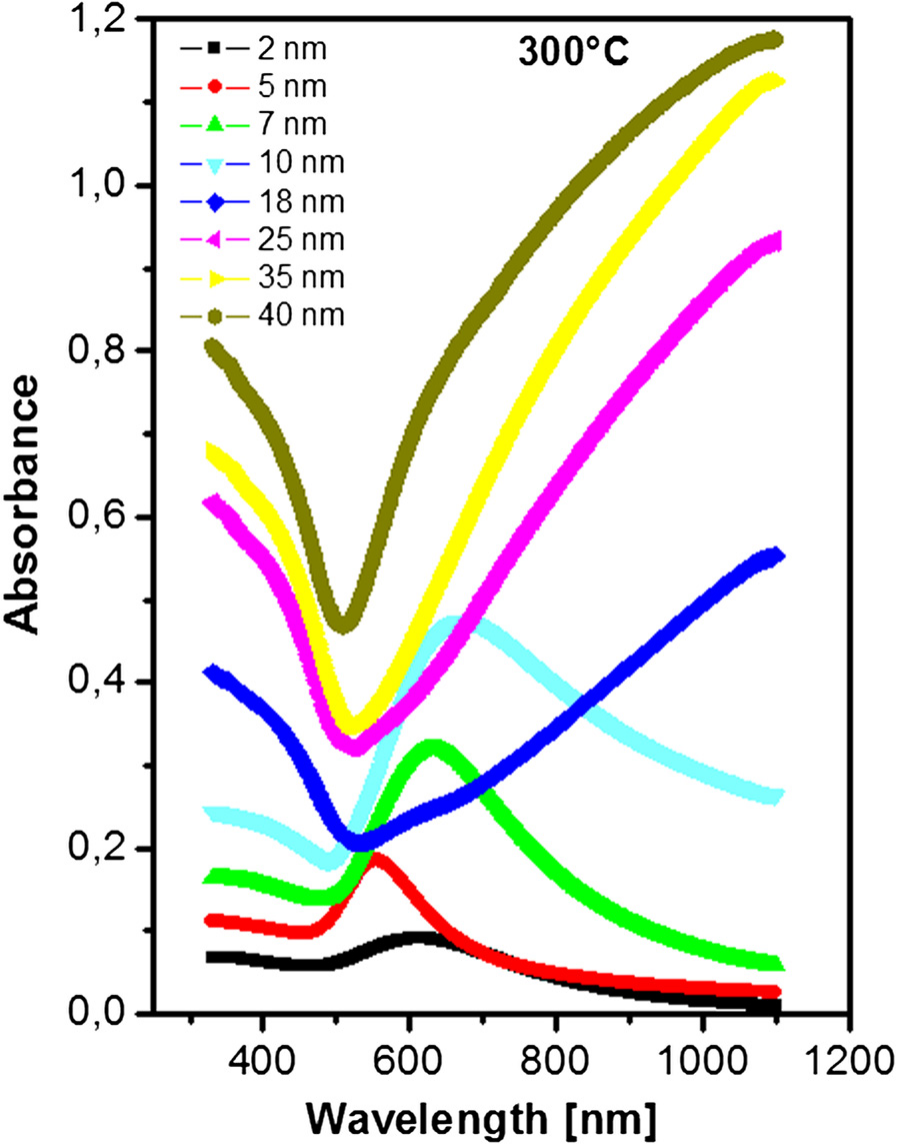
**UV–vis spectra of gold structures evaporated on glass heated to 300°C (300°C).** The numbers of the curves are Au thicknesses in nanometers.

The difference in absorbancies in extinction spectra of evaporated structures under RT and evaporated onto substrate heated to 300°C can be determined from Figure 6. The surface plasmon peak has been observed for the layer thickness up to 10 nm. The absolute value of the absorbance is higher in comparison to annealed structures, which is probably caused by the changes in structure morphology, density and size of Au clusters on the examined surface. The shift of the plasmon peak for lower thickness of Au was observed. This is probably caused by the interaction of gold nanoparticles, which may arise from a different mechanism of gold nanostructure growth when compared to the annealed one and when the layer is deposited on non-heated substrate.

Surface plasmon resonance (SPR) can be described as a collective oscillation of electrons in solid or liquid stimulated by incident light. The condition for the resonance appearance is established when the frequency of light photons matches the frequency of surface electrons oscillating against the restoring force of the positive nuclei. This effect when occurring in nanometer-sized structures is called localized surface plasmon resonance. Surface plasmons have been used to enhance the surface sensitivity of several spectroscopic measurements including fluorescence, Raman scattering, and second harmonic generation. Also, SPR reflectivity measurements can be used to detect molecular adsorption, such as polymers, DNA or proteins, and molecular interaction studies [24].

The shift of the curves in extinction spectra can be explained by the coupling of the electromagnetic field between surface plasmons excited in gold nanoparticles of different densities and sizes. The shift of surface plasmon resonance towards higher wavelengths has been observed for the gold nanolayer deposited on heated substrate to 300°C, while no shift has been observed for Au nanolayer deposited under RT and those consecutively heated. According to the shift in sheet resistance and different morphologies observed by atomic force microscopy, it can be concluded that for Au nanolayer deposited under 300°C, the insulating layer between gold nanoclusters causes shift of the surface plasmon resonance peak, as was observed e.g. in [25] for graphene and Au nanoparticles.

On the basis of the achieved results, it can be concluded that electrically continuous metal nanolayers with very low surface roughness can be prepared by evaporation on the substrate at elevated temperature. These structures also exhibit peaks of plasmon resonance up to Au thickness of 10 nm. The combination of surface plasmon resonance together with low surface roughness may find applications in the construction of biosensors for the detection of mycotoxins [26]. On the contrary, structures with different densities of gold nanoclusters prepared by the technique of evaporation at RT or consequently annealed can be of a great contribution for the construction of biosensors and DNA detection [27].

### Depth analysis

**Figure 7:**
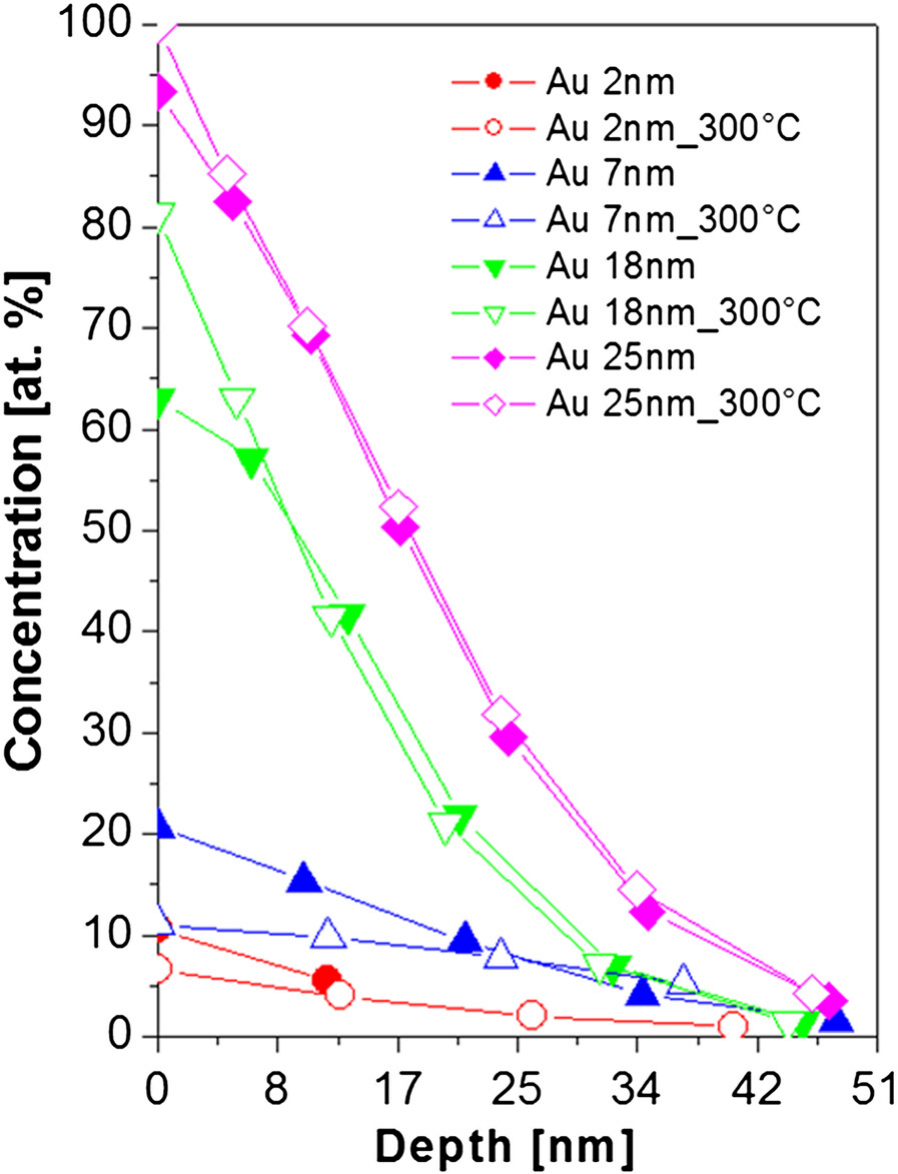
**RBS spectra of gold structures.** RBS spectra of gold structures evaporated on glass with room temperature and Au nanostructures evaporated on glass heated to 300°C (300°C).

The difference in surface metal distribution of evaporated structures under RT and evaporated onto substrate heated to 300°C is evaluated in Figure 7. The difference in the behavior of surface nanostructures in area on electrical discontinuity and continuity can be clearly seen. The electrically discontinuous layer exhibits significantly higher gold concentration when deposited on non-heated substrate. The heat treatment seems to be a positive promoter of surface diffusion (and nanocluster growth), mostly in the early stages of gold layer growth. This difference, thus, seems to affect the surface gold concentration; the higher the surface concentration, the more homogeneous the layer is. On the contrary, for higher gold thicknesses, when the layer is already electrically continuous, this difference is reversed. The influence of heated substrate causes the decrease of isolated nanocluster formation and thus positively influences its homogeneity. The isolated nanostructure, being less pronounced, increases the absolute gold concentration.

## Conclusions

The different surface properties of thermally annealed gold nanostructures in comparison to those evaporated onto heated substrate has been described. The heating of glass during the evaporation results in dramatic changes of the surface morphology and roughness. The substrate heating leads to the decrease of surface roughness for higher Au thickness, the electrical properties being also strongly influenced, the structure being more homogeneous. The electrically continuous layer is formed for the thicknesses above 20 nm. The shift of this threshold in comparison to structures evaporated and consequently annealed is probably caused by the surface diffusion in combination with local gold melting. The thermal annealing, on the contrary, leads to the creation of relatively large ‘spherolytic and hummock-like’ structures in the gold layer. The globular structure is strongly amplified by the thermal annealing probably due to local surface melting of gold nanoparticles during the process. The optical properties and appearance of peak of plasmon resonance for different thicknesses of Au structures are strongly influenced by prior glass heating.

## Competing interests

The authors declare that they have no competing interests.

## Authors’ contributions

AS carried out the sample preparation and participated on the AFM analysis and paper corrections. PS analyzed the surface morphology, evaluated the surface roughness and thickness, and designed the study. IK analyzed the electrical properties and carrier concentration of evaporated and annealed samples. PM and AM performed the RBS analysis. VŠ participated in the study coordination and paper correction. All authors read and approved the final manuscript.
